# Healthcare metaverse in surgery: scoping review

**DOI:** 10.1093/bjsopen/zrae155

**Published:** 2025-03-07

**Authors:** Benoit Jauniaux, Joshua Burke, Deena Harji

**Affiliations:** Department of Colorectal Surgery, Manchester University NHS Foundation Trust, Manchester, UK; Department of Colorectal Surgery, Manchester University NHS Foundation Trust, Manchester, UK; Leeds Institute of Medical Research, University of Leeds, Leeds, UK; Robotics and Digital Surgery Initiative, Royal College of Surgeons of England, London, UK; Department of Colorectal Surgery, Manchester University NHS Foundation Trust, Manchester, UK; Robotics and Digital Surgery Initiative, Royal College of Surgeons of England, London, UK; Clinical Trials Research Unit, Leeds Institute of Clinical Trials Research, University of Leeds, Leeds, UK

## Abstract

**Background:**

The metaverse is an emerging concept in surgery, with much interest in its highly immersive and interactive virtual environment. Despite the growing interest and importance in healthcare, the metaverse is still very much in its early phase of evolution and adoption in surgery, with debate on its definition and components. This scoping review provides a summary of the evidence and current understanding for the use of the metaverse in surgery.

**Methods:**

Embase and MEDLINE were searched using scoping review methodology with a systematic search strategy, identifying any study examining the role of the metaverse in surgery without time limitation. After data extraction, a narrative synthesis was conducted to identify the components of the metaverse employed within surgery and the domains in which they were applied.

**Results:**

Of 97 articles found through the initial search, 15 studies were eligible for inclusion. Most of the studies were expert opinion pieces (46.6%), urology was the most common specialty (33.3%), and all studies were published after 2020. Studies were widely heterogeneous in study design and outcomes varied. The surgical metaverse was used across four main domains: education (53%), training (80%), operations (67%), and surgical care (53%).

**Conclusion:**

Surgery is rapidly moving towards the age of the metaverse. There is great potential; however, evidence is lacking on its effectiveness and there are risks associated with its implementation. Institutions must learn how to understand and safely adopt the metaverse into their domains of education, training, operations, and surgical care.

## Teaser

The metaverse is an emerging concept in surgery, with much interest in its highly immersive and interactive virtual environment. Its applications in surgery are broad ranging, including education and training, surgical planning, and the delivery of surgical care.

## Introduction

Technological advancements over the past eight decades have led to huge societal changes, both within and outside of medicine. There has been adoption and evolution of a range of technologies, from primitive text-based chats to complex virtual worlds with their own populations and functioning economies^1^. Digital health has started to revolutionize the social dynamic of medicine and surgery by overcoming geographical barriers to deliver healthcare services and training^2–4^. The COVID-19 pandemic rapidly accelerated the growth of digital technologies designed to help the world adapt to prolonged social restriction^5–7^. In surgery, this fundamentally changed the conventional surgeon–patient interaction^5^ and stimulated increased interest in the concept of a healthcare metaverse^8,9^.

The metaverse, coined by Neal Stephenson in 1992^8^, is now described as a ‘virtual world that converges the physical and digital worlds to support immersive experiences and interoperable data exchange on a decentralized and secure network^4,9^. Whilst this technology has been predicted to disrupt healthcare delivery, innovation, and research, the metaverse is still very much in its early phase of evolution and adoption, with debate on its definition and components^10^. In surgery, several digital technologies are considered to be at various stages of the innovation cycle, including augmented reality (AR) and virtual reality (VR) for simulation, artificial intelligence (AI), blockchain (to provide accessible, safe, and global data), environmental sustainability products, and robotics^5,6,8,11^. AR and VR technologies are used routinely in select institutions for surgical training to replicate or simulate unique surgical procedures ahead of the index procedure^3^. The metaverse could amplify the use of these technologies by providing unprecedented access to AR/VR and create a real-time online space where all stakeholders can interact together within a virtual surgical community^12^. The potential applications of the metaverse in surgery are significant. The aim of this scoping review was to evaluate the literature on the use of the metaverse in surgery, including its current and future roles in the provision of surgical care and training.

## Methods

This review, protocol, and the corresponding data are reported in accordance with the PRISMA extension for scoping reviews (PRISMA-ScR) and with the PRISMA-ScR checklist^13,14^.

### Information sources and search strategy

**Fig. 1:**
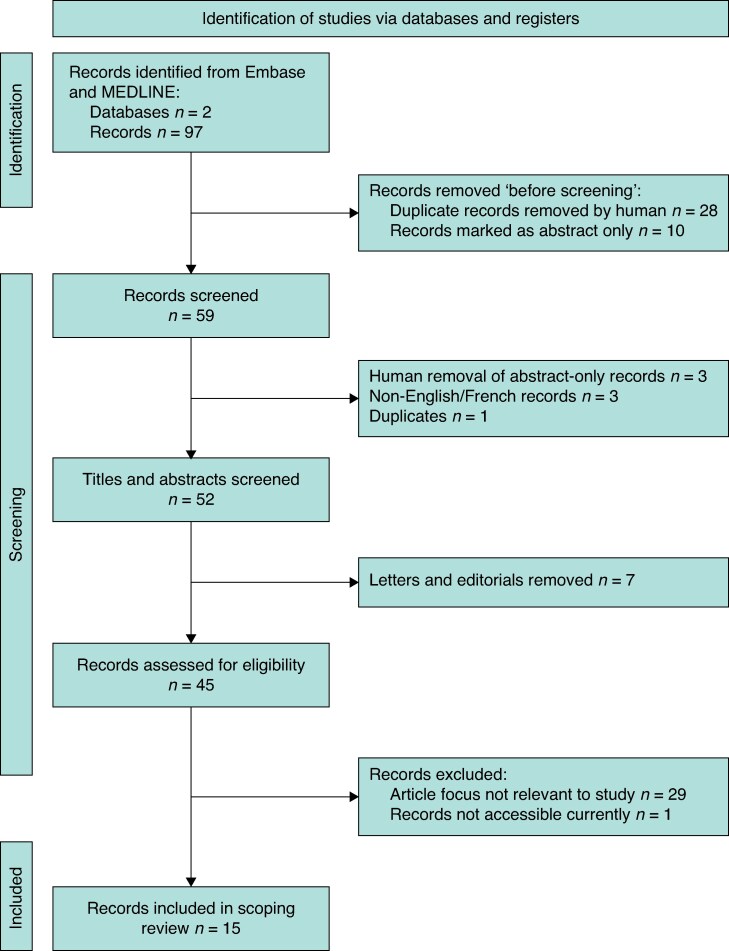
Flow diagram for PRISMA extension for scoping reviews

Embase and MEDLINE databases were searched systematically to identify records published without time limitation up to 1 February 2024. There are no Medical Subject Heading-specific terms for the metaverse; therefore, the keyword search string ‘Metaverse’ AND ‘Surg*’ was used. Duplicates and conference abstracts were excluded from identified English or French language original articles (*Fig. 1*). To ensure all data were captured and to identify any missed articles, citations and reference lists of selected studies were reviewed.

### Eligibility criteria

Inclusion criteria: accessible peer-reviewed scientific journal articles of any study design or published reports focusing on describing and/or evaluating and/or reviewing the metaverse in surgery; and written in English or French languages or translated into English or French languages.

Exclusion criteria: conference abstracts; and reports, letters, and editorials without original data.

### Study selection

All three authors (B.J., J.R.B., and D.H.) independently screened the titles and abstracts of all publications and excluded records that were not available in English, editorials or letters without original data, and records not related to metaverse and surgery domains. Data were extracted into Microsoft^®^ Excel (Microsoft, Redmond, WA, USA). Subsequently, two of the authors (B.J. and J.R.B.) independently assessed the content of the full text and resolved discrepancies through discussion with the senior author (D.H.).

### Data extraction and synthesis of results

The following data items were extracted: authors, year of publication, title, country, specialty, study type, metaverse definition, domains of metaverse applications to surgery, digital technology type and/or type of services, outcomes in relation to specific examples of the metaverse in surgery, and limitations. A narrative synthesis was conducted to identify the components of the metaverse employed within surgery and the domains in which they were applied. Descriptive data are expressed using basic statistics, including numbers and percentages. All data were entered into Microsoft^®^ Excel for analysis.

## Results

The initial search returned 97 references. After removal of duplicates, conference abstracts, letters and editorials without original data, and non-English articles, 45 remained for review (*Fig. 1*). Of these, 15 met the eligibility criteria and are included in the narrative synthesis (*Table S1*). The most common article type was expert opinion pieces (7 studies). There were seven primary research studies, prospectively examining the impact of the metaverse on surgery. Urology was the most common study focus (5 studies). One letter and one editorial were selected after meeting the criteria of including original data. Twenty nine studies were excluded, even when they described digital technologies such as VR and AR, when there was no mention or implication of how it could be applied to the metaverse and surgery. The earliest study was reported in 2021 and the majority of studies were published in 2023 (*Table S1*). Studies were conducted in various countries, with Italy being the most common country (6 studies).

### Definition

The definition of the metaverse varied across all articles (*Table 1*). The metaverse was referred to as a ‘digital world’ or a ‘virtual world’ by 50.0% of studies. The metaverse was divided into different categories by two studies. One study did not provide a definition for the metaverse. The definitions all encompass key components of the original definition of the metaverse, with a focus on virtual/digital worlds and immersive technologies.

**Table 1:** Definitions of the metaverse

Study author	Metaverse definition
Ammendola *et al*.^2^, 2023	A virtual shared reality where several worlds can be created, expanded, and explored from everywhere, both in a classical and in an immersive way
Ammendola *et al*.^6^, 2024	A digital world in which people can move through their own reproductions (avatars or digital twins) and interact in real time with other digital people
Checcucci *et al*.^15^, 2023	The next iteration of the Internet that uses AI, AR, VR, and increasingly connected networks, such as 5G, to create immersive and interactive online environments
Gandi *et al*.^16^, 2023	A parallel dimension in which the physical and virtual worlds merge enabling users to interact by using emerging technologies to enhance their actions and decisions Categorized into AR, mirror worlds, VR, and lifelogging
Gobeka *et al*.^17^, 2022	A virtual environment that combines AR, VR, and blockchain, as well as social media values, to imitate the real world
Gonzales-Romo *et al*.^18^, 2023	An alternative virtual space where humans can interact not only with each other but also with digital objects and has the potential to become an environment with benefits for medical education and training
Gouveia *et al*.^12^, 2023	Internet access via AR/VR/MR/XR, through a headset, and is already considered to be the next-generation mobile computing platform
Kim *et al*.^7^, 2023	A tool for enabling personalized digital therapeutics by connecting health providers and patients with tailored treatment in all areas of medical services
Koo^19^, 2021	A reproduction of reality in a virtual space
Matwala *et al*.^20^, 2023	The metaverse allows one to immerse themselves within the Internet A metaverse may combine VR and AR, or only use one of these The main characteristic is the ability to interact with other users of the metaverse in that moment, for example via personal avatars
Rahman *et al*.^21^, 2023	The fusion of the prefix ‘meta-’, signifying ‘beyond,’ and the term ‘universe’, encompassing a shared virtual space that emerges through the amalgamation of physical and digitally augmented realities
Randazzo *et al*.^22^, 2023	All definitions fall into one of these four categories: AR, lifelogging, mirror world, and VR
Sun *et al*.^9^, 2023	A digital virtual world in which people can live as a digital virtual identity
Tan *et al*.^4^, 2022	An interconnected online universe with the synergistic combination of VR, AR, and MR
Zattoni *et al*.^23^, 2023	Not defined

AI, artificial intelligence; AR, augmented reality; VR, virtual reality; MR, mixed reality; XR, extended reality.

### Components of the surgical metaverse

The key component metaverse digital technologies used in surgery were: VR (14 studies), AR (11 studies), AI (5 studies), technological innovations (8 studies), blockchain (3 studies), telementoring (7 studies), digital twins (5 studies), telemedicine (9 studies), telesurgery (4 studies), lifelogging (3 studies), photogrammetry (1 study), and gamification (1 study). Applications of these component digital technologies in surgery are defined in the glossary (*Table S2*) and are summarized with specific examples in different surgical specialties (*Table S1*).

### Applications of the surgical metaverse

**Fig. 2:**
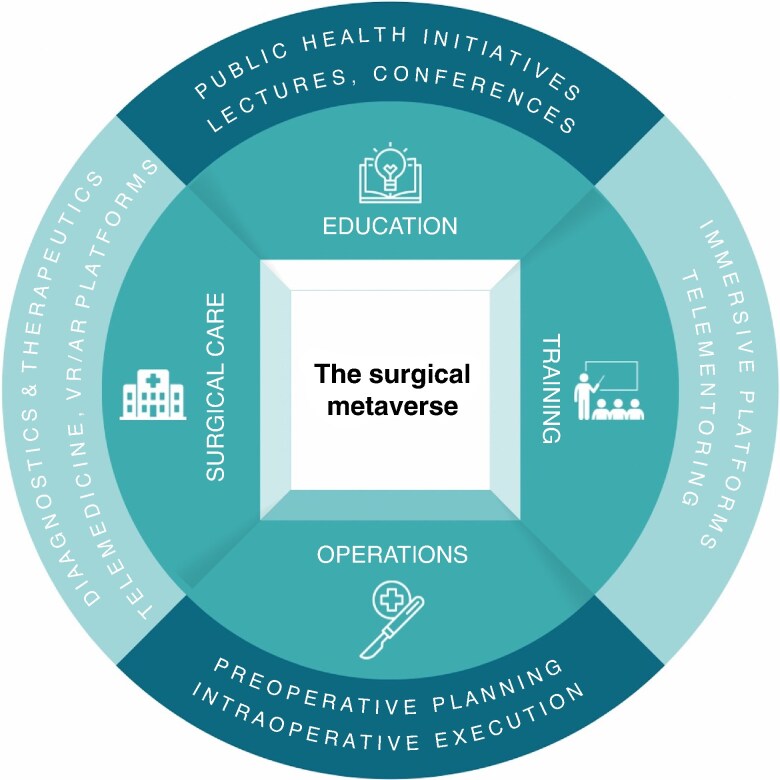
Domains of metaverse applications to surgery VR, virtual reality; AR, augmented reality.

The surgical metaverse was used across four domains (*Fig. 2* and *Table S1*): education (53%), training (80%), operations (67%), and surgical care (53%).

#### Domain 1: education

Eight studies applied the metaverse to deliver surgical education. The target audiences described were medical students (6 studies), surgical trainees (8 studies), and patients for public health education (5 studies). The metaverse facilitated remote student and trainee workshops in five studies^6,9,19,22,23^. VR and AR in the metaverse allowed virtual immersion into the anatomy of the human body, allowing students to experience a realistic view of anatomy and surgical approaches from remote settings^4,18^. Two ophthalmology and three urology studies described or demonstrated the immersive use and future potential of VR and AR in surgical trainee education^4,7,17,22,23^. Gobeka *et al*.^7^ found that metaverse characteristics such as VR, AR, and technological innovations could also be used to increase learning opportunities and enhance accessibility via three-dimensional (3D) videos through social media. In a pilot study, a virtual neurosurgery meeting space using photogrammetry-developed immersive cadaveric anatomy models was positively validated by surgical trainees who agreed the system should form part of formal neurosurgery training^18^. In the field of public and patient education, a recent review showed that VR is a feasible education tool and increases patients’ satisfaction, knowledge, and understanding^16,24^. In three separate studies, the use of lifelogging mobile applications and wearable devices or VR headsets was explored in the context of educating patients on urological conditions. The findings of these studies suggested that these technologies could enhance patient empowerment, whilst reducing the number of hospitalizations for investigations or monitoring^7,16,23^.

#### Domain 2: training

A review of 12 studies describing the metaverse in surgical training found telementoring as the principal application (7 studies). Ammendola *et al*.^2^ used telementoring to successfully supervise a robotic cholecystectomy and left colectomy from Dubai, with the operating surgeon in Italy, meeting in the ‘Metaverse Surgical Hospital, USA’. This same platform was later used to host a hepatopancreatobiliary online workshop^6^. Surgeons from all over the world, represented by their avatars, observed and supported the execution of a robotic liver resection. Digital twinning of patients for training was described by six studies, with AR technology providing hands-on simulation opportunities through its ability to replicate patients^4,16,22^. Technological innovations were also described in facilitating access to the metaverse in surgical training. Operations were viewed live and remotely by users globally in the metaverse, for training purposes^7,20^. In a cardiothoracic surgery conference, over 200 surgeons simultaneously attended a surgical training session using head-mounted displays and linking in with the smart operating room in Seoul National University Bundang Hospital^19^. Surgeons were able to interact with multiple 360° 3D cameras and sound technology built in the operating room.

#### Domain 3: operations

Ten studies described the role of the metaverse in operations, including some overlap between the different phases: pre-operatively (8 studies), intraoperatively (10 studies), and post-operatively (3 studies). In the case series of Checcucci *et al*.^15^, surgeons, represented by their digital avatars, met in a virtual room before surgery and participated in a virtual consultation on the surgical strategy for robotic partial nephrectomies with the use of digital twins. Robotic or laparoscopic procedures were then carried out according to the simulated surgical strategy. In 2021, 29 patients with a renal neoplasm underwent successful robot-assisted radical nephrectomy, across eight different hospitals, performed by a single operator located in the Qingdao tertiary referral hospital^25^. Intraoperatively, AR telestration was used successfully in breast surgery for non-invasive localization of impalpable tumours^12^. Three studies described the potential for postoperative remote home telehealth assessments and monitoring to reduce hospital stay and personalize rehabilitation^9,16,23^.

#### Domain 4: surgical care

Eight studies examined the surgical care applications of the metaverse. All studies described the implementation of telemedicine. It was used successfully as a transactional space to provide remote surgeon–patient consultations^26,27^, for immersive virtual meetings and conference platforms to facilitate management planning of patients^16,23^, and to organize postoperative teleclinics for plastic surgery patients^9^. Three studies explored examples of lifelogging or wearable devices^7,16,22^. Kim and Kim^7^ described various lifelogging apps and wearable devices to track or record and predict patterns in bladder dysfunction disorders.

## Discussion

**Fig. 3:**
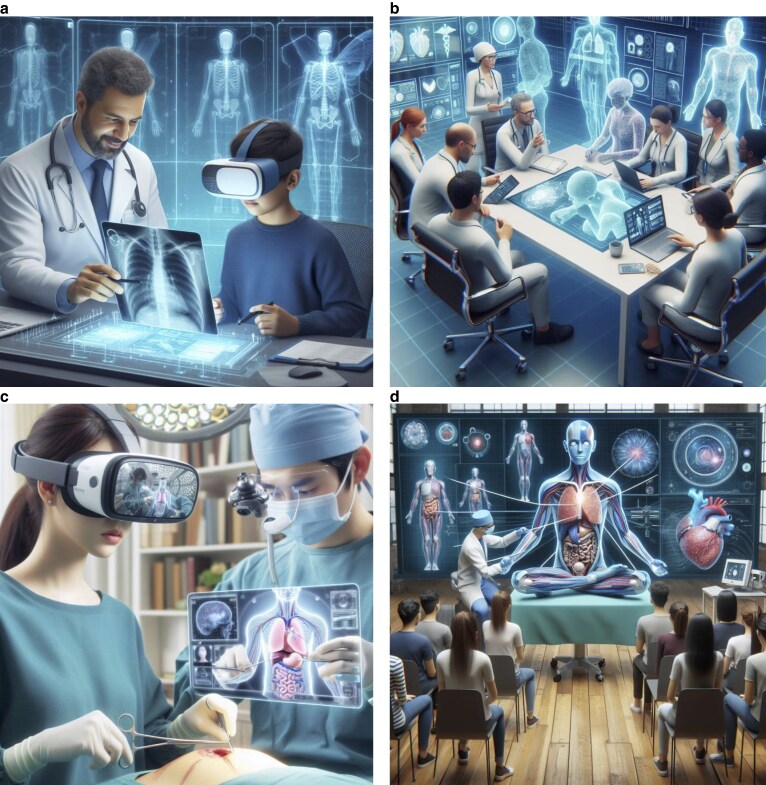
Illustrations of a surgical metaverse with virtual and augmented reality **a** A virtual consultation in the metaverse. **b** A multidisciplinary team meeting with participants connecting in the metaverse. **c** Telementoring. **d** A lecture/workshop in the metaverse. Source: authors, using Microsoft^®^ (2024) Image Creator (https://designer.microsoft.com/image-creator).

Surgery is rapidly moving towards the age of the metaverse. Since it became a popular term in healthcare over the past 3 years, research has started to highlight its current and potential role in medicine and surgery^28,29^. The World Economic Forum anticipates that digital innovation is an essential factor in future healthcare infrastructure and is required to decentralize and democratize healthcare^30^. Most studies focusing on the metaverse in surgery have been published in the past 18 months (*Table 1*). Despite the growing interest in the metaverse and its importance, there is a heterogeneous body of literature highlighting its role in surgery, and up-to-date evidence regarding its implementation and effectiveness is lacking. This evidence gap is behind other specialties such as emergency medicine^8^. However, recent studies in surgery propose the metaverse as an emerging technology, envisioned as the next evolution of the Internet and the future of healthcare; the metaverse is a crossroads of tactile and digital dimensions, facilitating real-time interactions, creating immersive and interactive online environments, and amalgamating the physical and digital worlds^15,21^. Several buzzwords are regularly used in descriptions of the metaverse, such as ‘VR’, ‘AR’, ‘AI’, ‘telemedicine’, ‘telementoring’, ‘technological innovations’, ‘blockchain’, ‘digital twins’, and ‘lifelogging’. These technology buzzwords are applicable to different categorical domains of the metaverse in surgery (*Fig. 3*).

The metaverse can bridge the educational gap between acquiring skills required for the real-world and training in a virtual environment. In medical schools, this could limit the requirement and costs of cadavers to teach anatomy^5,28^. Methods of surgical education are changing with the rapid evolvement of new technologies. Particularly with AR and VR, education in surgery could utilize the characteristics of the metaverse to increase freedom and provide a greater variety of personalized immersive and interactive learning environments for both surgical trainees and trainers^22^. One could imagine an online metaverse platform used to dissect virtual models of real organs, feel the consistency of the tissues, and even enter the interior of structures to evaluate their anatomical relationships (*Fig. 3*). However, in the current body of literature, there is insufficient evidence evaluating learning performance and effectiveness in comparison with traditional teaching methods. In patient education, the metaverse could improve doctor–patient communication and bolster public education on surgical conditions, individual health conditions, and upcoming surgical procedures^16^.

The metaverse has the potential to revolutionize surgical training, providing a world where reality and simulation are so harmoniously integrated that they are indistinguishable. The two main applications of the metaverse in training are immersive mirror worlds and telementoring. A mirror world is defined as a space representing a complex simulation of the real world^31^. Digital twins are explored by several studies and could be used for difficult or high-risk surgical operations to help predict a patient’s recovery, complications, and potential therapeutic alternatives^28^. AI could be implemented to introduce variation, as it happens in real life with real patients^22^. This technology is already beginning to develop^32^. A novice surgeon could consult in real time with a specialist surgeon elsewhere in the world on an immersive platform with patient-specific anatomy and details, both before surgery and during surgery, and create large data sets for in silico clinical trials^15,33,34^. The latter is known as telementoring and is regularly described as a concept within telemedicine where an expert physician guides another in a different geographical location^35^. This technology could reduce cost and distance inequalities regarding the exposure of tertiary centre trainees and smaller district hospitals to complex or rare cases^6^.

Technological innovations in the metaverse are beginning to directly affect the management of surgical patients both perioperatively and intraoperatively. Perioperatively, digital twinning technology allows surgeons to collaboratively plan precise surgical strategies for each patient^20^. AR and VR technologies are being developed to provide live graphics such as for optimal incision/trocar placement, without impairing a surgeon’s visibility^12,36^. With the development of fifth-generation (5G) technology, network speeds have greatly improved, supporting the advancement of telesurgery, which in turn reduces healthcare costs and increases accessibility^4^. Furthermore, the platforms used for simulation and training can reduce the risk of complications and errors during actual surgical procedures^37^. Other evolving digital technologies include consent and preoperative counselling for patients using AR and VR on metaverse platforms^20,21,38^. It is foreseeable that, in the future, patients could be given VR headsets and personalized digital avatars to interact with AI technologies in the metaverse, which could safely replace the need for surgeons to counsel patients before surgery, reducing the burden on staffing requirements^39^.

The metaverse can be applied to diagnostic and therapeutic domains for surgical patients and can be broadly separated into synchronous and asynchronous models of telemedicine^40^. Synchronous models involve virtual platforms for live patient–doctor consultations, whereas asynchronous models include triage tools or lifelogging technologies to record patient details via apps and wearable devices^4^. This could remove the boundaries of physical distance, whilst giving various experts simultaneous access to share information and interact, via their avatars, with 3D models of patient imaging and investigations. Synchronous telemedicine allows patients to see doctors remotely with a real-life experience^41,42^, to be used alongside traditional consultations. The metaverse could link real-time locations and objects utilized in the delivery of medical services to enhance teleconsultations. Virtual platforms could also be used for counselling or support groups where patients can interact with each other or staff via their own customized avatars^22,23^, increasing accessibility to psychological support for patients. Asynchronous telemedicine increases flexibility and has the potential to bypass the linear workflow of scheduled appointments in which bottlenecks are inevitable^4^. Examples include AI triage ophthalmology clinics to reduce emergency service burdens^4^ and lifelogging technology^7^.

Medicine and surgery have traditionally centred around human-to-human interactions. The technology revolution could be difficult for many patients to adapt to. With increasing amounts of data available virtually, cybersecurity is an issue and with a potential threat to patient confidentiality. Blockchain technology can be used to encrypt patient data in a decentralized manner to enable the storage and exchange of digital assets across platforms, enforcing compliance with medical standards in practices and processes^22,43,44^. On the other hand, if data are unlawfully acquired it could be very difficult to contain this data breach due to the decentralized nature of blockchain^9^. Blockchain, along with other requirements for telemedicine and telesurgery, requires efficient infrastructure and efficient 5G network settings, which are not currently available worldwide^12^. It is essential that the metaverse is implemented and distributed globally, along with 5G networks, to prevent wealth-gap and social inequalities regarding access to novel healthcare technology. The applications of the metaverse are still very much in the early experimental stages. The widespread adoption of these technologies requires robust evidence demonstrating cost-effectiveness, as well as appropriate infrastructure, which would require significant financial resources^12,15,20,23^. It is also important to consider the ecological costs of the production of hardware and the maintenance of blockchain technologies^16^. Further research is required to explore the sustainable impact of the metaverse on healthcare and the potential beneficial impacts of remote medicine with regard to reducing transport requirements. Ethical issues can arise, including biases in data and algorithms in technology such as representation in AR and digital avatars, which may lead to unfair or discriminatory outcomes in metaverse applications^3^. Developers must engage in discussions and research related to ‘algorethics’ to ensure that metaverse technologies are designed and applied in an ethically responsible manner^23^.

This scoping review has both strengths and limitations. It included a comprehensive search of the literature regarding the use of the metaverse across the field of surgery, with applications relevant to all surgical specialties. Using this approach builds on previous publications that explored the applications of the metaverse within a specific surgical specialty^22,45^ or a specific allied technology, for example telementoring^2,6^ or advanced imaging^19^. This provides a broader scope and application of the metaverse. The search strategy and methods are easy to repeat, meaning that future developments can be measured. The review has several limitations. First, the search was not very broad and only included two databases and two languages. The metaverse technology has identifiable limitations, with a narrow current application range and high heterogeneity between the study designs. While many studies shared commonalities, most were very different in methodologies and design, making them challenging to compare. There were limited primary data studies with original data and most of the selected studies were expert opinion pieces. Consequently, more robust evidence is needed to draw strong conclusions regarding the applications of the metaverse and their effectiveness within surgery. There is likely publication bias, with some studies only listing the positive impacts of the metaverse digital technologies described. The four main extracted domains of the metaverse in surgery may overlap in some details and concepts.

There is little doubt that digital technology and the metaverse will continue to grow as an essential contributor to medical healthcare and surgical practice. The potential appears unlimited at this stage; however, there are risks associated with its implementation. To reach its effective utility and reliability in the field of surgery, future developments and primary research are expected. Institutions must learn how to understand and implement the metaverse safely in the settings of education, training, operations, and surgical care. It is likely that, in the coming decades, developments will mean that the surgical metaverse will have more realism, cultural influence, and value, but this needs to be driven by the core needs of patients, healthcare provision, and the available resources.

## Supplementary Material

zrae155_Supplementary_Data

## Data Availability

The data sets generated during and/or analysed during the present study are available from the corresponding author upon reasonable request.
