# Supplementary material for: Healthcare metaverse in surgery: scoping review
Source: BJS Open. 2025 Mar 7;9(2):zrae155. doi: 10.1093/bjsopen/zrae155 (PMC11886832; doi:10.1093/bjsopen/zrae155)
Supplement: zrae155_Supplementary_Data [file zrae155_supplementary_data.zip › Supplementary Materials BJS_Open.docx]

**The Healthcare Metaverse in Surgery: Scoping Review**

*B Jauniaux^1^, JR Burke^1,2,^, D Harji^1,2,3^*

Benoit Jauniaux*,* ^1^Department of Colorectal Surgery, Manchester University NHS Foundation Trust, Manchester, UK*;* [benoit.jaunaux@doctors.org.uk](mailto:benoit.jaunaux@doctors.org.uk), ORCID ID 0000-0002-2527-2112

**Corresponding author:** Joshua Richard Burke, ^1^Leeds Institute Medical Research, University of Leeds, UK; ^2^ Robotics and Digital Surgery Initiative, Royal College of Surgeons of England, England; [joshburke@doctors.org.uk](mailto:joshburke@doctors.org.uk), ORCID ID 0000-0003-4027-747X

Deena Harji*,* ^1^Royal College of Surgeons of England, England, [deena_harji@hotmail.com](mailto:deena_harji@hotmail.com) ^2^Department of Colorectal Surgery, Manchester University NHS Foundation Trust, Manchester, UK, ^3^Robotics and Digital Surgery Initiative, ORCID ID 0000-0002-8493-3312

**Supplementary Materials – Index**

**Supplementary Figures & Tables:**

Supplementary Table S1: Characteristics of included studies (n = 15). – **Page 2**

Supplementary Table S2: Glossary of key component digital technologies used in surgery. – **Page 6**

**Table S1: Characteristics of included studies (n = 15).**

| Author, year, country, specialty | Study design | Domains of Metaverse in surgery | Digital technology described | Outcome | Limitations and/or further consideration |
| --- | --- | --- | --- | --- | --- |
| Ammendola et al. 2023,^(2)^, Italy, General | *Letter to the editor* (with original data) | Training, Operations | VR, Telementoring | - Virtual avatars - Cholecystectomy & left colectomy performed in Italy, supervised from Dubai - Platform: ‘Metaverse Surgical Hospital, USA’ | - Implementation - Lack of regulation regarding adoption of the Metaverse |
| Ammendola et al. 2024 ^(6)^, Italy, HPB | Cross-sectional | Training, Operations | VR, Telementoring, | - Supervised and observed robotic hepatectomy in an HPB surgery workshop at ‘Metaverse Surgical Hospital, USA. - Surgeons joined from all over the world with personal avatars for discussions | - N/a |
| Checcucci et al. 2023 ^(15)^, Italy, Urology | *Case series* | Operations | VR, AR, Digital twins, Telemedicine | - Metaverse platform with personal avatars - Urology surgical planning strategies discussed - Interactive and immersive 3D VR models of the patients’ kidneys prior to the procedures (n = 9) | - Costs to implement |
| Gandi et al. 2023 ^(16)^, Italy, Urology | Narrative review | Education, Training, Operations, Surgical Care | VR, AR, AI, Tech. innovations, Blockchain, Digital twins, Telemedicine, Telesurgery, Lifelogging | - Virtual surgery described - Applications to conferences and meetings - Lifelogging devices and applications for patients - Education of patients with VR | - Data security - Cultural and social access barriers - Ecological costs of hardware |
| Gobeka et al. 2022 ^(17)^, Turkey, Opthal. | *Cross-sectional* | Education | VR | - Social media in healthcare - Comparing 3D and 2D videos as educational tools for ophthalmology surgical trainees and the potential to integrate this with VR | - N/a |
| Gonzales-Romo et al. 2023 ^(18)^, USA, Neurosurg. | *Pilot single-arm case report* | Education, Training | VR, Photo-grammetry | - Virtual collaboration between neurosurgery trainees and instructors - Virtual anatomy and dissection can be demonstrated using photogrammetry and watched remotely live - 95% of residents agreed it should be part of the residency training | - Lack of evidence vs traditional methods of education |
| Gouveia et al. 2023 ^(12)^, Portugal, Breast | Cross- sectional | Training, Operations | AR, Telementoring | - Metaverse can make AR a practical surgical tool available worldwide by reducing travel costs, distance and time constraints - Telementoring example using AR-enhanced headsets e.g. live graphics of surgical incisional placement, without impairing visibility | - Implementation due to lack of infrastructure - Tech. issues e.g. 5G networks required |
| Kim et al. 2023 ^(7)^, Korea, Urology | Narrative review | Education, Training, Surgical Care | VR, AR, Tech. Innovations, Digital twins, Telemedicine, Lifelogging | - Mobile apps e.g. bladder diaries can be used to monitor urological disorders - Wearable devices for bladder dysfunction - Mobile pelvic floor trainers can communicate wirelessly with an app - Smart-toilets for diagnosis and management - AR and VR simulation/education on a Metaverse platform - Telemedicine cost-effective and high patient satisfaction with remote consultation in urology | - More clinical evidence for digital therapeutics is required - Increased compliance required for wearable devices |
| Koo., 2021 ^(19)^, Korea, Thoracic | *Editorial* (with original data) | Training | VR, AR, Tech. innovations | - Metaverse virtual thoracic surgery training conference - Personal avatars - Interactive lectures, discussions and live surgery observed. - Participants could focus on different views e.g. the view from the laparoscope - ‘Smart’ operating rooms with 360° VR cameras, a high-resolution camera, and fluorescent imaging equipment that can visualize lymph nodes in 1 place in the operating room - Play-back features | - N/a |
| Matwala et al. 2023 ^(20)^, Spain, Nonspecific | Narrative review | Training, Operations, Surgical Care | VR, AR, AI, Tech. Innovations, Telementoring, Digital twins, Telemedicine, Telesurgery | - Hundreds of users simultaneously accessing 3D first surgeon views of live operations - Telestration provides live overlaying annotations in the surgeon’s field of view -5 viewed remotely and live for real-time assistance, with easily accessible patient data e.g. recent imaging - AI characters could replace real people as trainers or to advise patients pre-operatively - Remote MDT’s - 3D VR surgical planning anatomical models could be viewed pre-operatively by multiple surgeons - Post-operative Teleclinics | - Confidentiality risks - High current cost to implementation |
| Rahman et al. 2023 ^(21)^, UK, Plastic | *Literature review* | Surgical Care | VR, AR, Tech. Innovations, Telemedicine | - Literature search ‘Metaverse in aesthetic and plastic surgery’ (n = 6) - Virtual consultations and virtual simulation - Empower consent with immersive virtual outcome journeys - Self-perception changing with appearance of digital avatars | - Ethical concerns over body image and mental well-being in relation to avatars |
| Randazzo et al. 2023 ^(22)^, Italy, Urology | Narrative review | Education, Training, Operations, Surgical Care | VR, AR, AI, Tech. innovations, Blockchain, Digital twins, Telemedicine, Lifelogging | - Immersive virtual environments for remote consultations, simulation, meetings and lectures - Long-term cost reductions and increased access to care - 3D modelling for urological surgical planning, training, or intraoperatively with AR and AI. - VR 3D platforms with personals avatar e.g. for counselling or support groups | - Blockchain difficulties for patient data encryption - Bias and stereotypes – lack of diversity |
| Sun et al. 2023 ^(9)^, China, Plastic | *Systematic review* | Education, Training, Operations, Surgical Care | VR, AR, Telementoring, Telemedicine, Telesurgery | - Systematic review ‘Metaverse in plastic surgery’ (n = 4) - Virtual operating rooms and avatars, virtual clinics, remote surgery, and virtual conferences - No time/distance constraints for consultations | - Difficulty recovering stolen data due to decentralisation - Renowned surgeons benefitting the most |
| Tan et al. 2022 ^(4)^, Singapore, Opthal. | Narrative review | Education, Training, Operations, Surgical Care | VR, AR, AI, Tech. innovations, Blockchain, Telementoring, Digital twins,  Telemedicine, Telesurgery, Gamification | - Ophthalmic teleconsultations reduce A&E pressures – triage tool - Personal avatars in virtual environment to improve realism of remote consultations - Applying AR and VR live for planning and guidance - AI could help triage cases - Blockchain for secure decentralised and exchangeable data - Digital twins of patients to improve simulation and precise surgery - Immersive public health education - Immersive use of AR and VR for visual loss and amblyopia therapy | - Requires reliable internet connectivity and secure platforms - Reduced in-person interactions - Cybersecurity risk |
| Zattoni et al. 2023 ^(23)^, Italy, Urology | *Mini review* | Education, Training, Operations, Surgical Care | VR, AR, AI, Tech. Innovations, Telementoring, Telemedicine | - MER headsets allow interactive 3D visualisation of patient anatomy pre-procedure, and intraoperatively with live essential information overlaid on the surgeon's view - MER for immersive and interactive education - Facilitate virtual therapy sessions - Facilitate convenient remote consultations, online scientific meetings and virtual support groups. - Remote patient monitoring to improve support for post-operative rehabilitation | - Expensive implementation of headsets - Wealth gap inequalities - AI technology requires developments |

Abbreviations: VR = virtual reality; HPB = Hepato-Pancreato-Biliary; AR = augmented reality; AI = artificial intelligence; 3D = three-dimensional; MDT = multi-disciplinary team; A&E = accident and emergency; MER = medical enhanced VR/AR.

| Component | Summary definition |
| --- | --- |
| Artificial Intelligence (AI) | - Computer systems performing various tasks at the level of or beyond the complexity of that historically only humans could do, including communication, data processing, storing and formatting, generating, supervised and unsupervised learning, and prediction ^(39)^ |
| Algorethics | - The field of ethics that focuses on the development and deployment of algorithms to ensure that they are transparent, accountable, and fair ^(23)^ |
| Augmented Reality (AR) | - AR superimposes and overlays virtual images onto the real environment, enhancing the users perception and interaction with the physical environment ^(20, 21)^ |
| Blockchain | - A distributed database to encrypt data records, called blocks, in a transparent and traceable way ^(16)^ |
| Digital twins | - A digital mirrored representation which the behaviour and interaction of a physical asset with other physical assets ^(4)^ |
| Gamification | - Incorporating in-game conditional rewards for completing specific tasks ^(4)^ |
| Lifelogging | - Using smart devices to augment the physical world by capturing and recording daily activities, which are then stored and accessible through the internet ^(22)^ |
| Photogrammetry | - A technique allowing 3D objects to be simulated using coordinates and spatial measurements which are obtained from photographs of the objects ^(18)^ |
| Telementoring | - The provision of real-time guidance to a surgeon from an expert residing in a different geographical location ^(4)^ |
| Telestration | - A technique that allows users to annotate images or videos by drawing on them ^(12)^ |
| Telesurgery | - When surgery is remotely performed on a patient by a surgeon residing in a different location ^(4)^ |
| Virtual Reality (VR) | - A completely immersive and interactive experience in which someone is placed into a virtual environment ^(20)^ |

**Table S2: Glossary of key component digital technologies used in surgery.**

Abbreviations: VR = virtual reality; AR = augmented reality; AI = artificial intelligence.
